# Supplementary material for: Integrative functional analysis uncovers metabolic differences between Candida species
Source: Commun Biol. 2022 Sep 26;5:1013. doi: 10.1038/s42003-022-03955-z (PMC9512779; doi:10.1038/s42003-022-03955-z)
Supplement: Supplementary file 2 — Supplemental Material [file 42003_2022_3955_MOESM2_ESM.pdf]

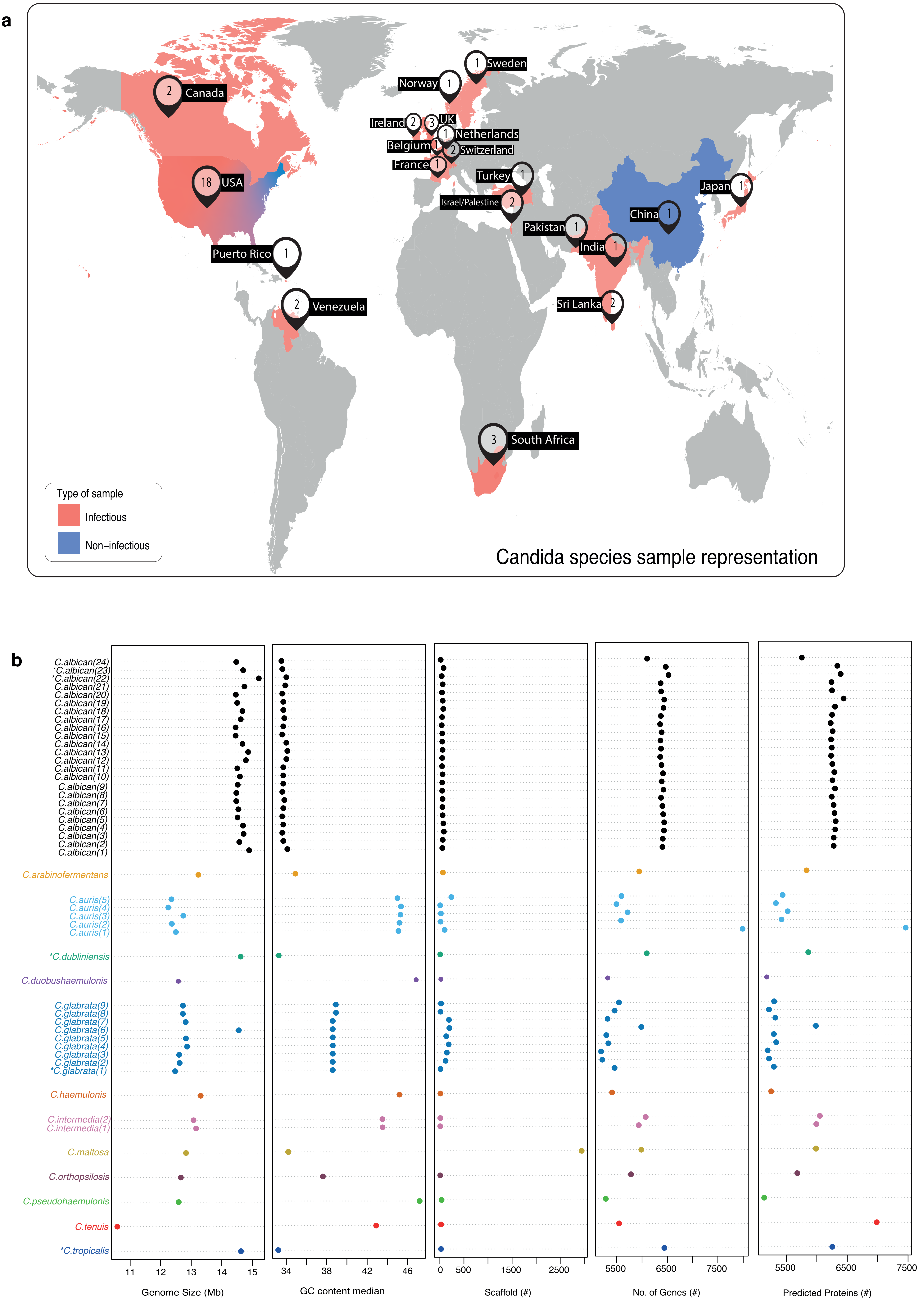

Supplementary Figure 1. a, Map of *Candida* species collection. Indication of global representation of samples. b, Sequence quality assessment. Genome size variation, GC content, number of contig and outcome of quality of the sequence.

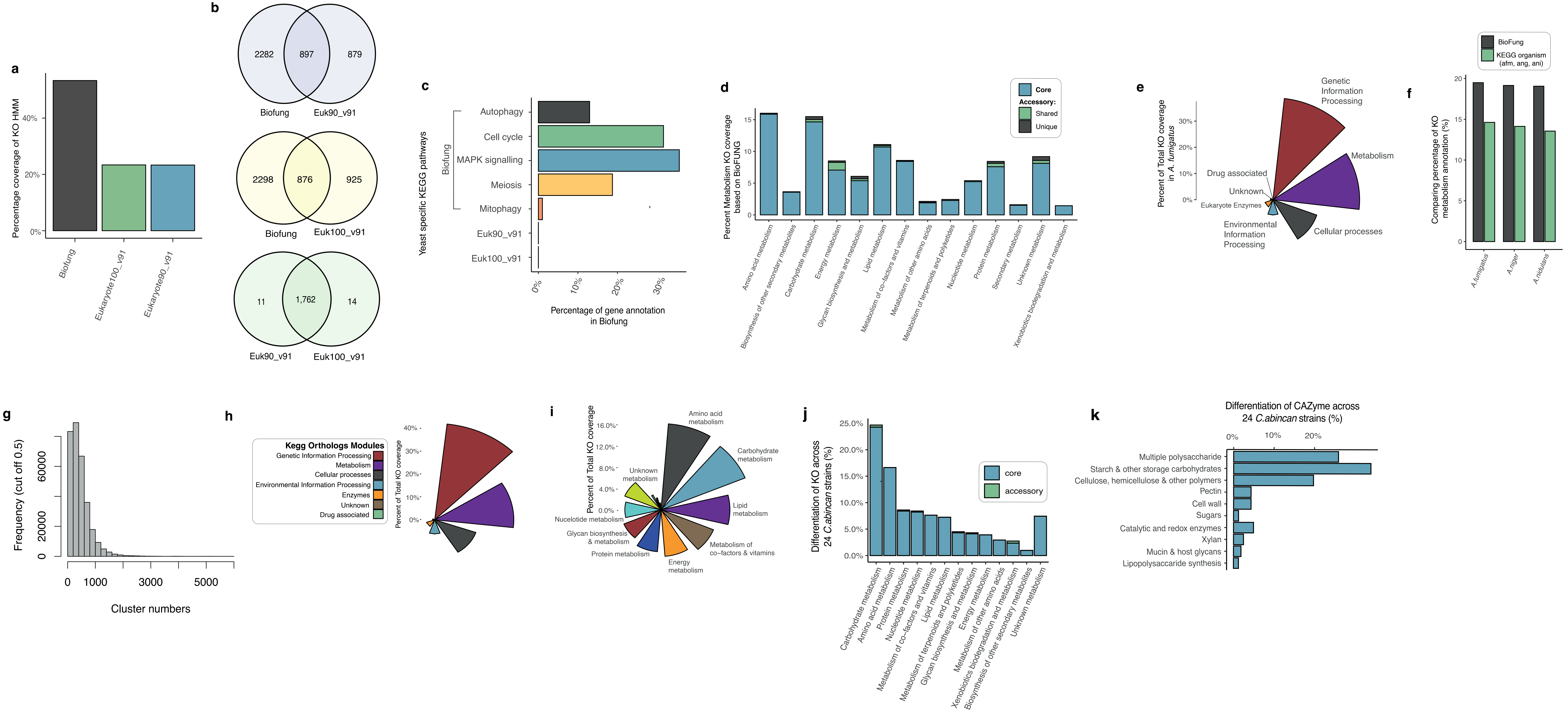

Supplementary Figure 2. a, Comparison of BioFung to available euk90 and euk100 KEGG profiles. Euk90_v91 and euk100_v91 are versions of pre-trained hmm for eukaryote database[61,62]. Coverage of BioFung was higher than of available hmm. b, Venn diagram displays the overlap of annotation. The overlap between BioFung vs euk90_v91, BioFung vs euk100_v91 and comparison of both eukaryote profiles from all *Candida* gene annotation. BioFung still has an enormous scope of unique KO, and small differences between eukaryote profiles can be observed. c, Validation assessing coverage of the yeast-specific pathway. We performed pathway analysis of KO annotations containing yeast-specific cell pathways. Observation of no yeast specific pathways was found in eukaryote annotation. d, Core and accessory overview of the metabolic pathway across *Aspergillus* strains using BioFung. Shared genome refers to features not present in all strains and unique genome features is exhibited by only one *Aspergillus* species denoting accessory functions. e, Coverage of BioFung annotation of *Aspergillus* fumigatus with similar function reportage of living cells. f, Annotation comparison of BioFung and KEGG for Aspergillus species. g, Uclust quality assessment by looking at the frequency of cluster numbers at 0.5 cut-offs. The cut-ff was inclusive of signature clusters capture and a broad range of core clusters. h, Core percentile coverage of functional annotation. First instance, distribution of KEGG module categories reflect functions seen in most microbiota species. Illustrates the extensive functional capability of *Candida* within each species. i, Core KEGG metabolism exploration in *Candida* species. Amino acid, carbohydrate and lipid metabolism contributes the greatest make up of gene function in *Candida* species. This indicates the contribution of different metabolic potential from *Candida*. j, Intra-strain metabolism analysis of *C. albicans* indicating genome differentiation. k, Clustering of 24 *C. albicans* strains of Carbohydrate Active enzymes demonstrating indistinguishable changes in genome.

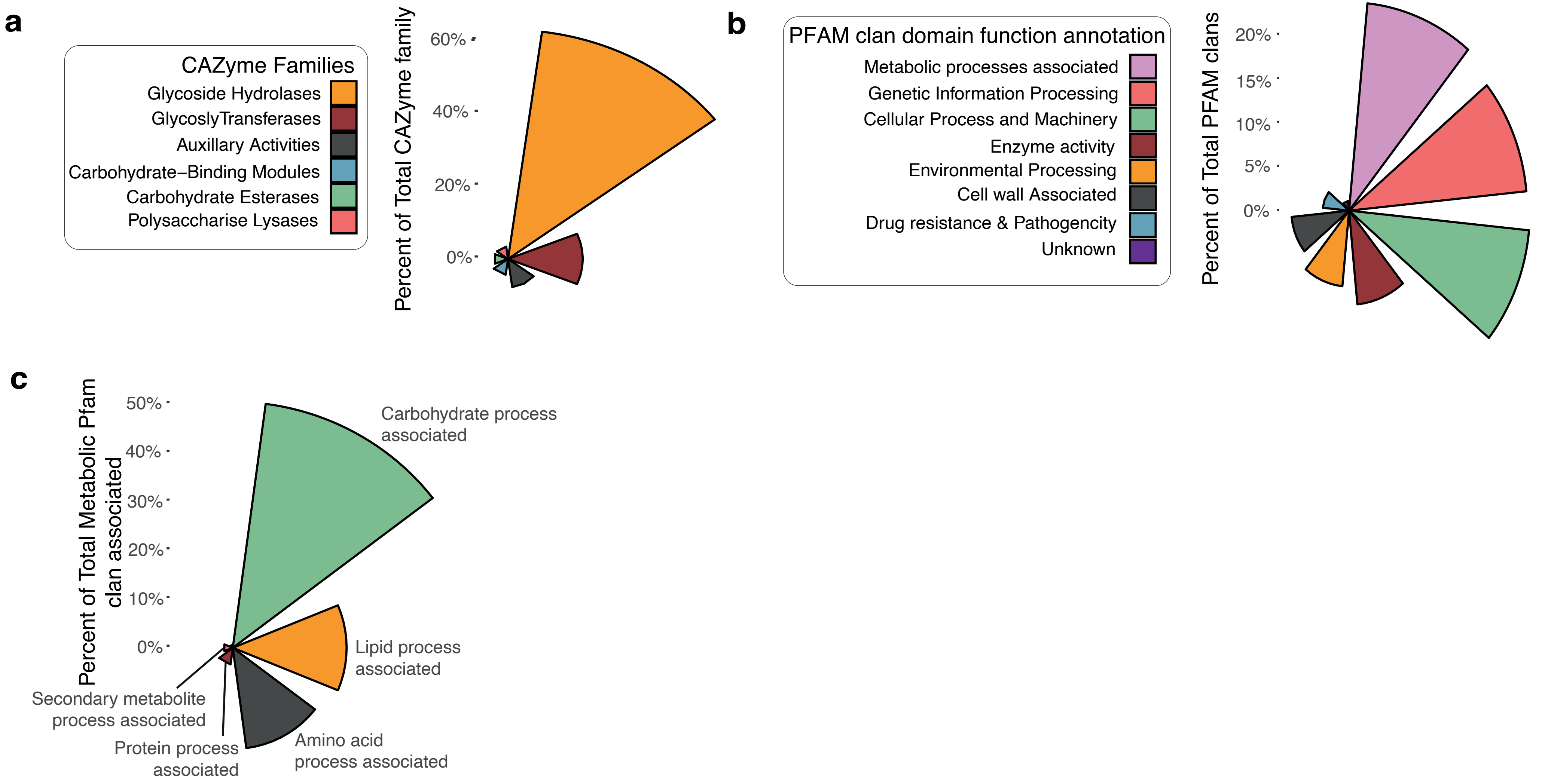

Supplementary Figure 3 a, Coverage of CAZyme annotation. Glycoside hydrolase family is the largest contributor in all *Candida* species. b, Coverage of Pfam annotation. Pfam coverage indicates that metabolic processes, genetic information processing and cellular process & machinery provide the immense repertoire of protein function in all *Candida* species. c, Core Pfam metabolic process analysis in *Candida* species. Carbohydrate, amino acid, and lipid-associated processes are the largest metabolomic protein function in *Candida* strains.

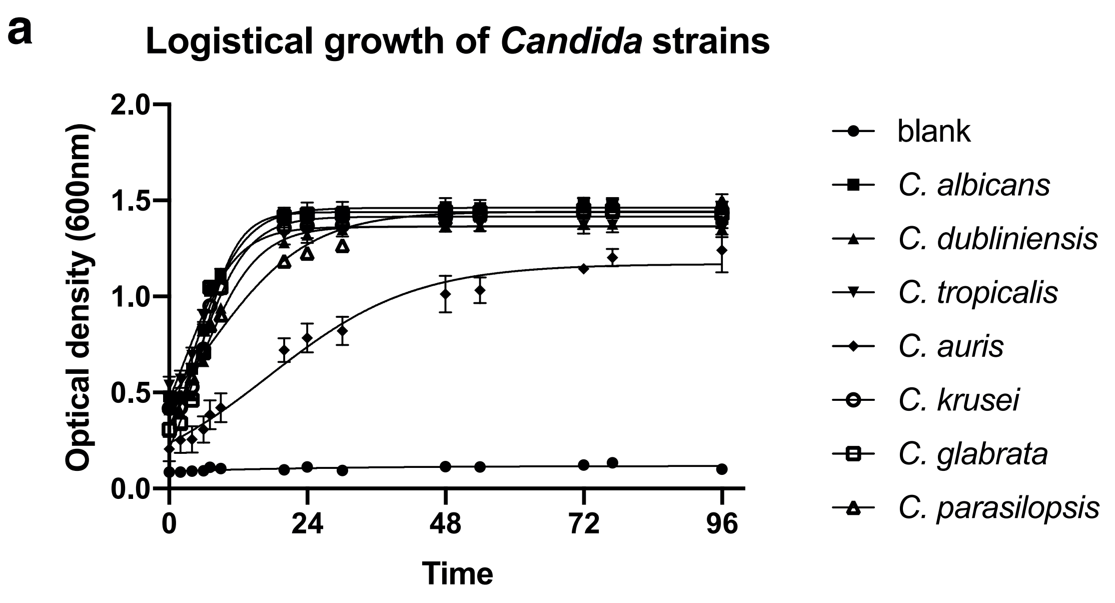

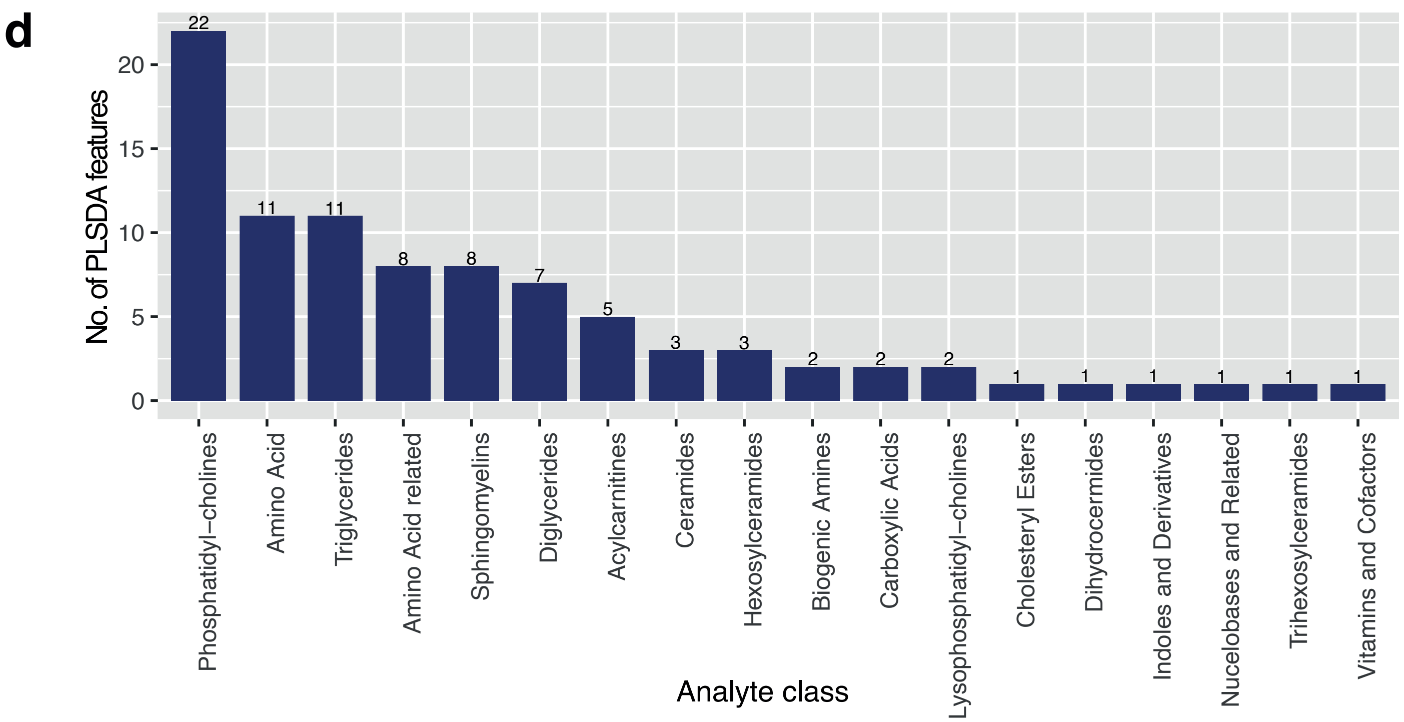

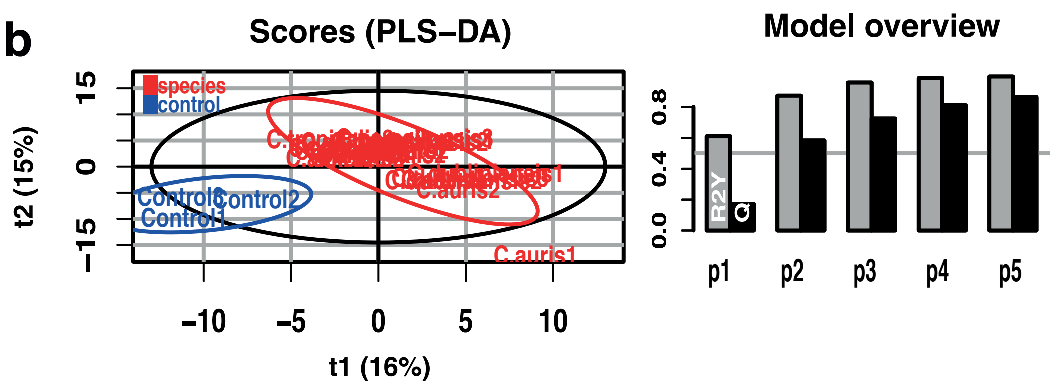

Control vs Species

PLSDA	R2X	R2Y(cum)	Q2(cum)	RMSEE	pre	pR2Y	pQ2
Total	0.524	0.997	0.864	0.019	5	0.2	0.05

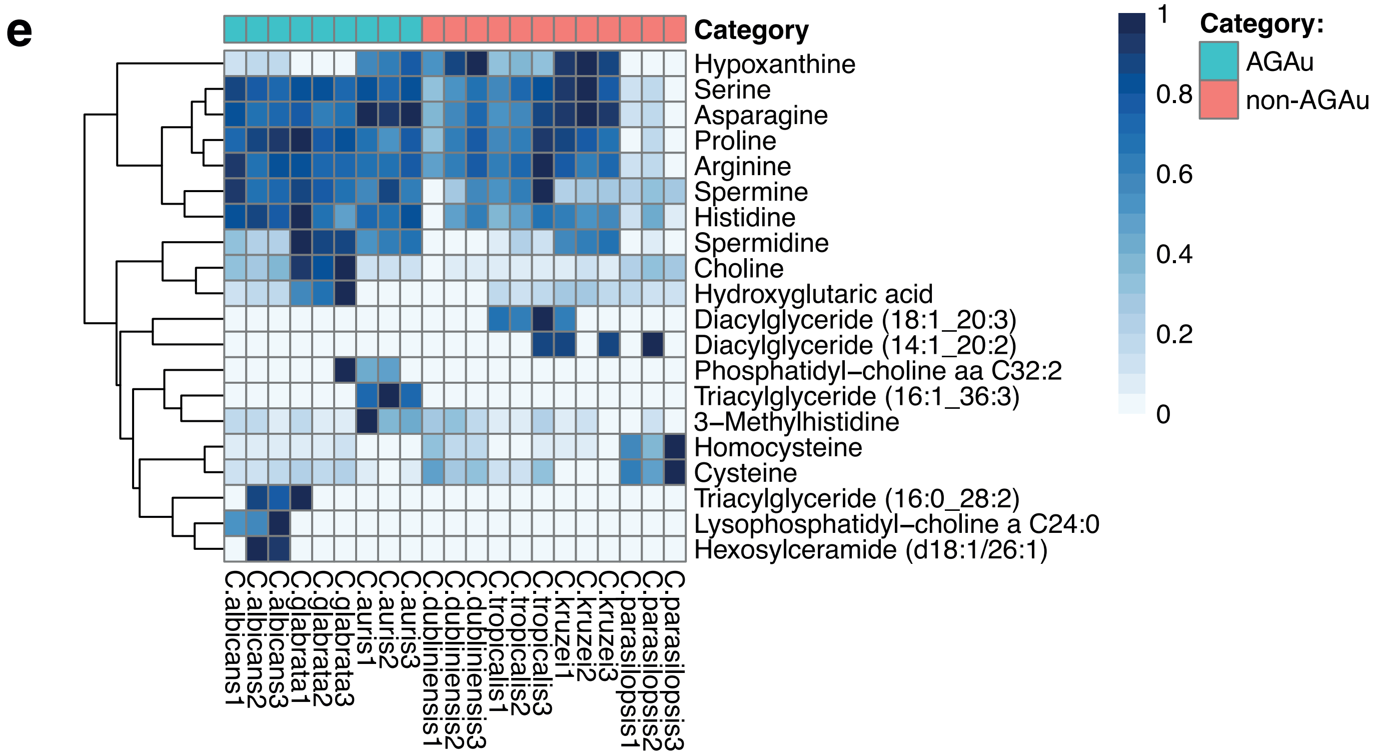

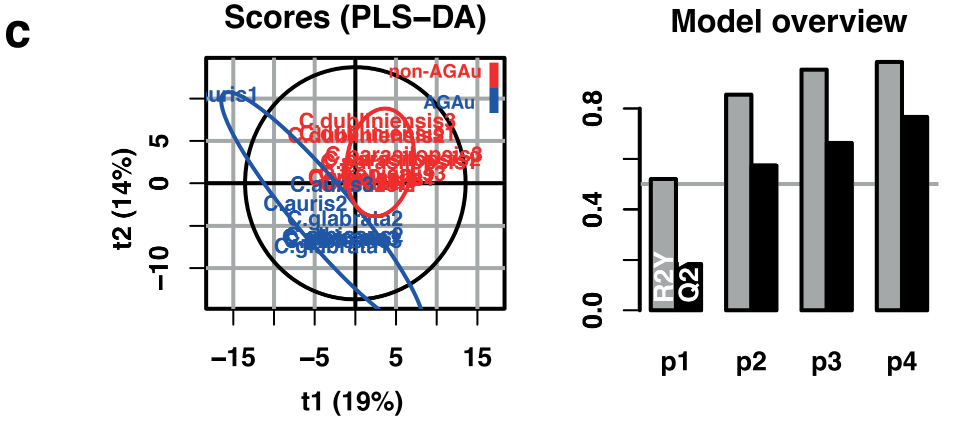

Invasive vs Non-invasive

PLSDA	R2X	R2Y(cum)	Q2(cum)	RMSEE	pre	pR2Y	pQ2
Total	0.501	0.984	0.766	0.071	4	0.3	0.05

Supplementary Figure 4 a, Growth curve of strains grown in the laboratory. All the strains were grown, and timepoint measurement of OD1 at 600nm was taken to obtain the mid-exponential phase (n=3 biologically replicates; error bars indicate (+/-)S.D. of triplicate measurement). b, Targeted metabolomics with PLS-DA analysis differentiating from media. PLS-DA score indicated a precise fitting performance of clustering media distinctly from Candida strains. c, Targeted metabolomics PLS-DA analysis based on AGAu and non- AGAu species. PLS-DA score plot indicated a fitting and predictive performance (2 latent variables, $R^2X = 0.501$, $R^2Y = 0.984$, $Q^2 = 0.76$). d, Analyte Class component features in PLS-DA analysis. A significant number of differential components between invasive and non-invasive strains. e, Heatmap of significant metabolites. Identified from PLS-DA, top 20 metabolites differentiated in AGAu and non-AGAu based on Wilcoxon rank-sum test ($P<0.05$).

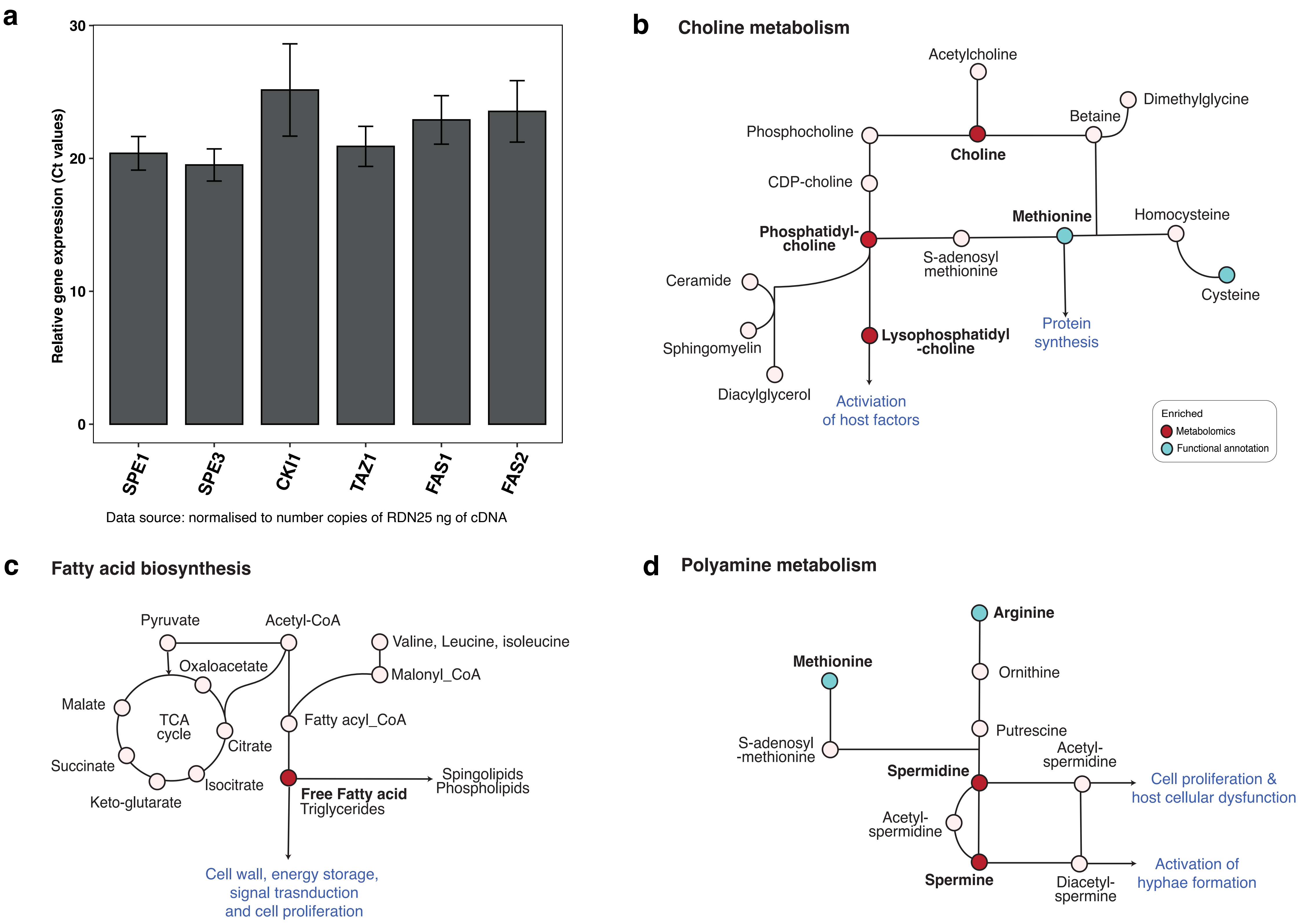

Supplementary Figure 5 a, Quantitative RT-qPCR validation of pathways with C. albicans. Indicating pathway genes (SPE1, SPE3, CK1, TAZ1, FAS1, FAS2) compared to their relative expression in Candida albicans, representative of AGAu species cluster. (n = 3 biological replicates; display the relative RNA expression level (Ct value); error bars indicate (+/-)S.D. of triplicate measurement). b, Choline metabolic pathway. Concise choline metabolism and its affect. c, Fatty acid metabolism. Concise fatty acid biosynthesis metabolism and its affect. d, Polyamine metabolism. Concise polyamine metabolism and its affect.

Supplementary Table 1, Information on database collection of 49 *Candida* strains. In-depth data collection of strain retrieval from a database, including strain id, genome id, sample type, origin country, year of collection, annotation availability.

No.	Sample name	Genome assembly	Genome ID	ENA ID	Biosample ID	Strain	Sequencing platform	Included as Refseq	Year	Orign	Sample type	Annotations available	Reference ID:
1	candida_auris_gca_001189475	gca_001189475	ASM118947v1	PRJNA267757	SAMN03200169	6684	Illumina HiSeq; Illumina MiSeq	3464668	2015	India	Urine	P,U	PMID: 26346253
2	candida_auris_gca_002759435	gca_002759435	Cand_auris_B8441_V2	PRJNA328792	SAMN05379624	B8441	PacBio	N/A	2017	Pakistan	Urine/blood	no results	PMID: 27988485
3	candida_auris_gca_002775015	gca_002775015	Cand_auris_B11221_V1	PRJNA328792	SAMN05379609	B11221	PacBio	9493448	2017	South Africa	patient (unknown location)	no results	PMID: 27988485
4	candida_auris_gca_003013715	gca_003013715	Cand_auris_B11220	PRJNA328792	SAMN05379608	B11220	PacBio	N/A	2017	Japan	Ear	no results	PMID: 27988485
5	candida_auris_gca_003014415	gca_003014415	Cand_auris_B11243	PRJNA328792	SAMN05379619	B11243	Illumina	N/A	2018	Venezuela	blood	no results	PMID: 30559369
6	candida_duobushaemulonis_gca_002926085	gca_002926085	CanDuoHae_v1.0	PRJNA421966	SAMN08161465	B09383	PacBio	8186598	2018	USA	foot ulcer	no results	PMID: 30559369
7	candida_glabrata_gca_000002545	gca_000002545	ASM254v2	PRJNA13831	SAMEA3138172	CBS 138	3700 Genetic Analyser/Licor 4200L DNA sequencer	354578	2002	Unknown	faecal	K,P,E,KO,U	PMID: 12527359
8	candida_glabrata_gca_001466525	gca_001466525	ASM146652v1	PRJNA297263	SAMN04121073	1A	Illumina MiSeq	N/A	2015	Norway	blood	P,U	WGS Project: LLWO01
9	candida_glabrata_gca_001466535	gca_001466535	ASM146653v1	PRJNA297263	SAMN04121074	1B	Illumina MiSeq	N/A	2015	Norway	blood	K,P,E,KO,U	WGS Project: LMAA01
10	candida_glabrata_gca_001466565	gca_001466565	ASM146656v1	PRJNA297263	SAMN04121078	3B	Illumina MiSeq	N/A	2015	Norway	blood	K,P,E,U	WGS Project: LMAB01
11	candida_glabrata_gca_001466575	gca_001466575	ASM146657v1	PRJNA297263	SAMN04121076	2B	Illumina MiSeq	N/A	2015	Norway	blood	P,U	WGS Project: LLZY01
12	candida_glabrata_gca_001466635	gca_001466635	ASM146663v1	PRJNA297263	SAMN04121075	2A	Illumina MiSeq	N/A	2015	Norway	blood	P,U	WGS Project: LLZZ01
13	candida_glabrata_gca_001466685	gca_001466685	ASM146668v1	PRJNA297263	SAMN04121077	3A	Illumina MiSeq	N/A	2015	Norway	blood	P,U	WGS Project: LMAY01
14	candida_glabrata_gca_002219185	gca_002219185	ASM221918v1	PRJNA374542	SAMN06328411	DSY562	PacBio	N/A	2014 (1995)	Switzerland	Oral	no results	PMID: 28663342

15	candida_glabrata_gca_002219195	gca_002219195	ASM221919v1	PRJNA374542	SAMN06328412	DSY565	PacBio	N/A	2015 (1995)	Switzerland	Oral	no results	PMID: 28663342
16	candida_haemulonis_gca_002926055	gca_002926055	CanHae_1.0	PRJNA421961	SAMN08161437	B11899	PacBio	9255618	2018	Israel	leg ulcer	no results	PMID: 30559369
17	candida_intermedia_gca_900106115	gca_900106115	CBS 141442 assembly	PRJEB14359	SAMEA4029010	CBS 141442	single-molecule real-time (SMRT) sequencing		2016	Sweden	wheat	P,U	PMID: 28385851
18	candida_intermedia_gca_900106125	gca_900106125	PYCC 4715 assembly	PRJEB14359	SAMEA4029009	PYCC 4715	single-molecule real-time (SMRT) sequencing		2016	Sweden	sewage in Portugal	P,U	PMID: 28385851
19	candida_pseudohaemulonis_gca_003013735	gca_003013735	Cand_pseudohaemulonii_B12108	PRJNA438484	SAMN08714088	B12108	Illumina	6426098	2015	Venezuela	patient (unknown location)	no results	PMID: 30559369
20	candida_tenuis_atcc_10573_gca_000223465	gca_000223465	Candida tenuis v1.0	PRJNA33673	SAMN00715317	ATCC 10573	454; Sanger	895348	2011	USA	trees, roots and beetles	K,P,KO,U	PMID: 21788494
21	candida_albicans_12c_gca_000773845	gca_000773845	Cand_albi_12C_V2	PRJNA75209	SAMN00767974	12C	Illumina	N/A	2014	USA	vaginal	no results	WGS Project: AJIQ01
22	candida_albicans_19f_gca_000775445	gca_000775445	Cand_albi_19F_V2	PRJNA75221	SAMN01048008	19F	Illumina	N/A	2014	USA	vaginal	no results	WGS Project: AJIV01
23	candida_albicans_ca6_gca_000784695	gca_000784695	Cand_albi_Ca6_V2	PRJNA120431	SAMN03164130	Ca6	Illumina	N/A	2014	Sweden	blood	no results	WGS Project: AJJG01
24	candida_albicans_gc75_gca_000773735	gca_000773735	Cand_albi_GC75_V1	PRJNA75223	SAMN00767984	GC75	Illumina	N/A	2014	South Africa	oral (healthy)	no results	WGS Project: AJIW01
25	candida_albicans_l26_gca_000775455	gca_000775455	Cand_albi_L26_V2	PRJNA75211	SAMN01048004	L26	Illumina	N/A	2014	USA	vaginal	no results	WGS Project: AJIR01
26	candida_albicans_p34048_gca_000775465	gca_000775465	Cand_albi_P34048_V1	PRJNA75229	SAMN01048010	P34048	Illumina	N/A	2014	Turkey	blood	no results	WGS Project: AJIZ01
27	candida_albicans_p37005_gca_000773745	gca_000773745	Cand_albi_P37005_V2	PRJNA75217	SAMN01048006	P37005	Illumina	N/A	2014	USA	oral (healthy)	no results	WGS Project: AJIU01
28	candida_albicans_p37037_gca_000773825	gca_000773825	Cand_albi_P37037_V1	PRJNA75231	SAMN01048011	P37037	Illumina	N/A	2014	USA	oral (healthy)	no results	WGS Project: AJJA01
29	candida_albicans_p37039_gca_000784515	gca_000784515	Cand_albi_P37039_V1	PRJNA75233	SAMN00767975	P37039	Illumina	N/A	2014	USA	blood	no results	WGS Project: JSXQ01
30	candida_albicans_p57055_gca_000775505	gca_000775505	Cand_albi_P57055_V1	PRJNA75239	SAMN01048013	P57055	Illumina	N/A	2014	USA	blood	no results	WGS Project: AJJB01

31	candida_albicans_p57072_gca_000773805	gca_000773805	Cand_albi_P57072_V1	PRJNA75227	SAMN00767978	P57072	Illumina	N/A	2014	USA	blood	no results	WGS Project: AJIY01
32	candida_albicans_p60002_gca_000784525	gca_000784525	Cand_albi_P60002_V1	PRJNA75219	SAMN01048007	P60002	Illumina	N/A	2014	USA	blood	no results	WGS Project: JSXP01
33	candida_albicans_p75010_gca_000784575	gca_000784575	Cand_albi_P75010_V1	PRJNA75235	SAMN00769059	P75010	Illumina	N/A	2014	Belgium	blood	no results	WGS Project: JSXR01
34	candida_albicans_p75016_gca_000784595	gca_000784595	Cand_albi_P75016_V1	PRJNA75237	SAMN01048012	P75016	Illumina	N/A	2014	Israel	blood	no results	WGS Project: JPEV01
35	candida_albicans_p75063_gca_000775525	gca_000775525	Cand_albi_P7506_V1	PRJNA75241	SAMN01048014	P75063	Illumina	N/A	2014	France	blood	no results	WGS Project: AJJC01
36	candida_albicans_p76055_gca_000784505	gca_000784505	Cand_albi_P76055_V1	PRJNA75243	SAMN01048016	P76055	Illumina	N/A	2014	USA	blood	no results	WGS Project: AJJD01
37	candida_albicans_p76067_gca_000784495	gca_000784495	Cand_albi_P76067_V1	PRJNA75245	SAMN01048017	P76067	Illumina	N/A	2014	Canada	blood	no results	WGS Project: AJJE01
38	candida_albicans_p78042_gca_000784615	gca_000784615	Cand_albi_P78042_V1	PRJNA75247	SAMN01048015	P78042	Illumina	N/A	2014	USA	blood	no results	WGS Project: JPEW01
39	candida_albicans_p78048_gca_000773725	gca_000773725	Cand_albi_P78048_V1	PRJNA75225	SAMN01048009	P78048	Illumina	N/A	2014	Canada	blood	no results	WGS Project: AJIX01
40	candida_albicans_p87_gca_000774085	gca_000774085	Cand_albi_P87_V2	PRJNA75215	SAMN00767982	P87	Illumina	N/A	2014	South Africa	oral/vaginal	no results	WGS Project: AJIT01
41	candida_albicans_p94015_gca_000773755	gca_000773755	Cand_albi_P94015_V1	PRJNA75213	SAMN01048005	P94015	Illumina	N/A	2014	USA	blood	no results	WGS Project: AJIS01
42	candida_albicans_sc5314_gca_000784635	gca_000784635	Cand_albi_SC5314_V4	PRJNA120009	SAMN01041717	SC5314	Illumina	N/A	2014	USA	Patient (unknown location)	K,P,KO,U	PMID: 15239821
43	candida_albicans_sc5314_gca_000784655	gca_000784655	Cand_albi_SC5314_V3	PRJNA191536	SAMN01041717	SC5314	Illumina	N/A	2014	USA	Patient (unknown location)	K,P,KO,U	PMID: 15239821
44	candida_albicans_wo_1_gca_000149445	gca_000149445	ASM14944v2	PRJNA16373	SAMN02953609	WO-1	ABI/Sanger technology	N/A	2009(1986)	USA	blood	P,U	PMID: 19465905
45	candida_arabinofermentans_nrrl_yb_2248_gca_001661425	gca_001661425	Canar1	PRJNA207879	SAMN00794644	NRRL YB-2248	Illumina	N/A	2016(1998)	USA	unidentified wood boring insect larvae	P,U	PMID: 27535936
46	candida_dubliniensis_cd36_gca_000026945	gca_000026945	ASM2694v1	PRJEA34697	SAMEA2272258	CD36	Sanger	101528	2009(1995)	Ireland	oral	P,E,K,KO,U	PMID: 19745113

47	candida_maltosa_xu316_gca_000344705	gca_000344705	Cmaltosa_v01	PRJNA184737	SAMN02981501	Xu316	Illumina HiSeq	N/A	2013(2010)	China	Laboratory (Unknown)	no results	PMID: 20668703
48	candida_orthopsilosis_co_90_125_gca_000315875	gca_000315875	ASM31587v1	PRJEA83665	SAMEA2272376	Co 90-125	Roche GS FLX instrument	1011928	2012(1928)	Puerto Rico (Latin America)	diarrhea(blood)	K,P,KO,U	PMID: 22563396
49	candida_tropicalis_mya_3404_gca_000006335	gca_000006335	ASM633v3	PRJNA13675	SAMN02953608	MYA-3404	ABI	6369408	2009(1994)	Holland	blood	K,P,E,KO,U	PMID: 19465905

* annotation column abbreviation expansion: K=KEGG genome annotation;C= ;P=PFAM annotation;E=eggnog annotation; KO=kegg ortholog (Phylogenomic); Z= CAZyme annotation; U=Uniprot annotation.

Supplementary Table 2, AGAu strain categorisation. Based on sample names, grouping, location of sample extraction and reference for strain.

No.	Sample name	Cluster species	Location of sample extraction	Reference
1	candida_auris_gca_001189475	AGAu	Urine	DOI: 10.1186/s12864-015-1863-z
2	candida_auris_gca_002759435	AGAu	Urine/blood	DOI: 10.1093/cid/ciw691
3	candida_auris_gca_002775015	AGAu	patient (unknown location)	DOI: 10.1093/cid/ciw691
4	candida_auris_gca_003013715	AGAu	Ear	DOI: 10.1093/cid/ciw691
5	candida_auris_gca_003014415	AGAu	blood	DOI: 10.1038/s41467-018-07779-6
6	candida_duobushaemulonis_gca_002926085	Non-AGAu	foot ulcer	DOI: 10.1038/s41467-018-07779-6
7	candida_glabrata_gca_000002545	AGAu	faecal	DOI: 10.1016/s0014-5793(02)03749-3
8	candida_glabrata_gca_001466525	AGAu	blood	DOI: 10.1128/CMR.12.1.80
9	candida_glabrata_gca_001466535	AGAu	blood	DOI: 10.1128/CMR.12.1.80
10	candida_glabrata_gca_001466565	AGAu	blood	DOI: 10.1128/CMR.12.1.80
11	candida_glabrata_gca_001466575	AGAu	blood	DOI: 10.1128/CMR.12.1.80
12	candida_glabrata_gca_001466635	AGAu	blood	DOI: 10.1128/CMR.12.1.80
13	candida_glabrata_gca_001466685	AGAu	blood	DOI: 10.1128/CMR.12.1.80
14	candida_glabrata_gca_002219185	AGAu	Oral	DOI: 10.1534/g3.117.042887
15	candida_glabrata_gca_002219195	AGAu	Oral	DOI: 10.1534/g3.117.042887
16	candida_haemulonis_gca_002926055	Non-AGAu	leg ulcer	DOI: 10.1038/s41467-018-07779-6
17	candida_intermedia_gca_900106115	Non-AGAu	wheat	DOI: 10.1128/genomeA.00138-17
18	candida_intermedia_gca_900106125	Non-AGAu	sewage in Portugal	DOI: 10.1128/genomeA.00138-17
19	candida_pseudohaemulonis_gca_003013735	Non-AGAu	patient (unknown location)	DOI: 10.1038/s41467-018-07779-6
20	candida_tenuis_atcc_10573_gca_000223465	Non-AGAu	trees, roots and beetles	DOI: 10.1073/pnas.1103039108
21	candida_albicans_12c_gca_000773845	AGAu	vaginal	DOI: 10.1534/genetics.104.033167
22	candida_albicans_19f_gca_000775445	AGAu	vaginal	DOI: 10.1534/genetics.104.033167
23	candida_albicans_ca6_gca_000784695	AGAu	blood	DOI: 10.1534/genetics.104.033167
24	candida_albicans_gc75_gca_000773735	AGAu	oral (healthy)	DOI: 10.1534/genetics.104.033167
25	candida_albicans_l26_gca_000775455	AGAu	vaginal	DOI: 10.1534/genetics.104.033167
26	candida_albicans_p34048_gca_000775465	AGAu	blood	DOI: 10.1534/genetics.104.033167
27	candida_albicans_p37005_gca_000773745	AGAu	oral (healthy)	DOI: 10.1534/genetics.104.033167
28	candida_albicans_p37037_gca_000773825	AGAu	oral (healthy)	DOI: 10.1534/genetics.104.033167
29	candida_albicans_p37039_gca_000784515	AGAu	blood	DOI: 10.1534/genetics.104.033167
30	candida_albicans_p57055_gca_000775505	AGAu	blood	DOI: 10.1534/genetics.104.033167
31	candida_albicans_p57072_gca_000773805	AGAu	blood	DOI: 10.1534/genetics.104.033167
32	candida_albicans_p60002_gca_000784525	AGAu	blood	DOI: 10.1534/genetics.104.033167
33	candida_albicans_p75010_gca_000784575	AGAu	blood	DOI: 10.1534/genetics.104.033167
34	candida_albicans_p75016_gca_000784595	AGAu	blood	DOI: 10.1534/genetics.104.033167
35	candida_albicans_p75063_gca_000775525	AGAu	blood	DOI: 10.1534/genetics.104.033167

36	candida_albicans_p76055_gca_000784505	AGAu	blood	DOI: 10.1128/mBio.01205-18
37	candida_albicans_p76067_gca_000784495	AGAu	blood	DOI: 10.1534/genetics.104.033167
38	candida_albicans_p78042_gca_000784615	AGAu	blood	DOI: 10.1128/mBio.01205-18
39	candida_albicans_p78048_gca_000773725	AGAu	blood	DOI: 10.1534/genetics.104.033167
40	candida_albicans_p87_gca_000774085	AGAu	oral/vaginal	DOI: 10.1128/mBio.01205-18
41	candida_albicans_p94015_gca_000773755	AGAu	blood	DOI: 10.1101/gr.174623.114
42	candida_albicans_sc5314_gca_000784635	AGAu	Patient (unknown location)	DOI: 10.1186/gb-2004-5-7-230
43	candida_albicans_sc5314_gca_000784655	AGAu	Patient (unknown location)	DOI: 10.1186/gb-2004-5-7-230
44	candida_albicans_wo_1_gca_000149445	AGAu	blood	DOI: 10.1101/gr.174623.114
45	candida_arabinofermentans_nrrl_yb_2248_gca_001661425	Non-AGAu	unidentified wood boring insect larvae	DOI: 10.1023/a:1001799607871
46	candida_dubliniensis_cd36_gca_000026945	Non-AGAu	oral	DOI: 10.1099/00221287-144-4-829
47	candida_maltosa_xu316_gca_000344705	Non-AGAu	Laboratory (Unknown)	DOI: 10.1111/j.1365-2672.2006.02915.x
48	candida_orthopsilosis_co_90_125_gca_000315875	Non-AGAu	diarrhea(blood)	DOI: 10.1371/journal.pone.0035750
49	candida_tropicalis_mya_3404_gca_000006335	Non-AGAu	blood	DOI: 10.1038/nature08064

Supplementary Table 3, Significant of functional annotation in enriched or depleted in AGAu group. Some annotations are statistically significant with the odds ratio of falling within the scale of 0 and 1. They have not been included in the figure but remain relevant. These have been captured in this table for CAZyme, KO and Pfam.

Cazyme	Cell wall substrate conversion	Reference for cazyme assay	Reference fungal cell wall
CBM43	Glucan	DOI: 10.1128/EC.00107-10	DOI: 10.1074/jbc.M807990200
GH1	Glucan	DOI: 10.1093/database/bar020	DOI: 10.1186/1471-2164-15-6
GH109	Glucan	DOI: 10.1038/nbt1298	DOI: 10.1016/j.celrep.2019.02.090
GH13	Glucan	DOI: 10.1007/s10930-010-9260-6	DOI: 10.1186/1471-2164-15-6
GH16	Glucan	DOI: 10.1111/cmi.12630	DOI: 10.1186/1471-2164-15-6
GH17	Glucan	DOI: 10.3390/jof4010018	DOI: 10.1186/1471-2164-15-6
GH2	Chitin/Glucan	Withers, S., "Glycoside Hydrolase Family 2" in *CAZypedia*, available at URL https://www.cazypedia.org/index.php/Glycoside_Hydrolase_Family_2, accessed 7 Feburary 2021.	DOI: 10.1186/1471-2164-15-6
GH27	Glucan	DOI: 10.1093/glycob/cwq105	DOI: 10.1186/1471-2164-15-6
GH3	Glucan	DOI: 10.1038/s41598-020-58015-5	DOI: 10.1186/1471-2164-15-6
GH39	Glucan	DOI: 10.1074/jbc.M117.789008	DOI: 10.1186/1471-2164-15-6
GH5	Glucan	DOI: 10.1128/EC.00187-10	DOI: 10.1186/1471-2164-15-6
GH51	Glucan	DOI: 10.1074/jbc.M117.789008	DOI: 10.1186/1471-2164-15-6
GH72	Glucan	DOI: 10.1042/BJ20110405	DOI: 10.1186/1471-2164-15-6
GH74	Glucan	DOI: 10.1016/S2095-3119(20)63254-3	DOI: 10.1186/1471-2164-15-6; DOI: 10.1007/s00253-019-09983-w
GH81	Glucan	DOI: 10.1111/cmi.12630	DOI: 10.1186/1471-2164-15-6
GH9	Glucan	DOI: 10.1126/science.1244273	DOI: 10.1104/pp.105.072652
GT57	Glucan	DOI: 10.3390/foods8100452	DOI: 10.1111/tpj.14417; DOI: 10.3390/jof4010006
GT66	Glucan	DOI:10.1074/jbc.M111.339036	DOI: 10.1111/tpj.14417; DOI: 10.1099/mic.0.067967-0
CE1	Pectin	DOI: 10.1016/j.biori.2017.02.001	DOI: 10.1186/1471-2164-15-785
CE12	Pectin	DOI: 10.1186/s13068-016-0518-x	DOI: 10.1186/1471-2164-15-785
GH105	Pectin	DOI: 10.1128/AEM.02114-18	DOI: 10.1186/1471-2164-15-6
GH43	Pectin	DOI: 10.1186/1471-2164-13-321	DOI: 10.1186/1471-2164-15-6
GH78	Pectin	DOI: 10.1128/AEM.00895-19	DOI: 10.1186/1471-2164-15-6

GH88	Pectin	DOI: 10.3389/fmicb.2019.00649	DOI: 10.1186/1471-2164-15-6
GH92	Pectin	DOI: 10.1111/j.1574-6941.2012.01381.x	DOI: 10.1016/j.celrep.2019.02.090
GH93	Pectin	DOI: 10.1073/pnas.1701130114	DOI: 10.1186/1471-2164-15-6
GT32	Pectin	DOI: 10.1186/1471-2180-11-198	DOI: 10.1016/j.celrep.2019.02.090
GT83	Pectin	DOI: 10.1016/j.jbiotec.2019.10.011	DOI: 10.1186/1471-2164-15-6
PL10	Pectin	DOI: 10.1111/j.1574-6941.2012.01381.x	DOI: 10.1186/1471-2164-15-9
PL4	Pectin	DOI: 10.1111/tpj.14417	DOI: 10.1186/1471-2164-15-6
PL4_1	Pectin	DOI: 10.1007/s10295-018-2098-1	DOI: 10.3390/ijms19082379
PL4_2	Pectin	DOI: 10.1111/tpj.14417	DOI: 10.3390/ijms19082380
PL4_4	Pectin	Hu, H. (2019). The Antimicrobial Defence and Biomass Decomposition of Fungus-Growing Termites and Microbial Symbionts. Department of Biology, Faculty of Science, University of Copenhagen.	DOI: 10.3390/ijms19082381
PL4_5	Pectin	Theobald, S. Genus-level studies of gene dynamics for the Aspergillus genus. (2018).	DOI: 10.3390/ijms19082382
GH115	Mannan	DOI: 10.1186/s12896-015-0154-8	DOI: 10.1186/1471-2164-15-6; DOI: 10.1186/s12896-015-0154-8
GH125	Mannan	DOI: 10.1038/nature13995	DOI: 10.1371/journal.pone.0116269
GH130	Mannan	DOI: 10.1074/jbc.M115.681460	DOI: 10.1074/jbc.M115.681460
GH36	Mannan	DOI: 10.1002/1873-3468.12250	DOI: 10.1186/1471-2164-15-6
GH38	Mannan	DOI: 10.1038/nature13995	DOI: 10.1186/1471-2164-15-6; DOI: 10.1007/s00253-019-09983-w
GH5_7	mannan	DOI: 10.1007/s00425-013-2005-y	DOI: 10.1186/1471-2164-15-37
GT62	Mannan	DOI: 10.1093/nar/gkt1178	DOI: 10.3390/jof4010006; DOI: 10.3390/jof4010006
GT71	Mannan	DOI: 10.1093/nar/gkt1178	DOI: 10.3389/fmicb.2018.01581
CBM50	Chitin	DOI: 10.1007/s00294-014-0471-9	DOI: 10.1007/s00294-014-0471-9
GH18	Chitin	DOI: 10.1186/1471-2180-9-184	DOI: 10.1186/1471-2164-15-6
GH20	Chitin	DOI: 10.1007/s00294-015-0530-x; DOI: 10.1128/EC.00022-15	DOI: 10.1186/1471-2164-15-6
GH76	Chitin/Mannan	DOI: 10.3389/fmicb.2019.02294	DOI: 10.1186/1471-2164-15-6
GT2_Chitin_synth_1	Chitin	PMID: 13475355	DOI: 10.1016/j.celrep.2019.02.090
GT2_Chitin_synth_2	Chitin	PMID: 13475356	DOI: 10.1016/j.celrep.2019.02.091
GH85	Fungal cell wall	DOI: 10.1093/database/bar020	DOI: 10.1016/j.cell.2016.10.043

*Core CAZyme present in all *Candida* cell wall enzymatic assay references (Fig. 1h)

Supplementary Table 4, Additional CAZymes that were significantly enriched in AGAu species, these were not included in the main figure.

CAZYME	p_value	Odd_ratio	Annotation	Direction
GH114	0.00113894	0	Multiple polysaccharide	Enriched
GH145	0.00909359	0	Sugars	Enriched
GH36	0.00909359	0	Cell wall	Enriched
GH43_30	0.00013329	0	Xylan	Enriched
GH43_33	0.00013329	0	Xylan	Enriched
GH5_24	0.00013329	0	Cellulose, hemicellulose & other polymers	Enriched
GH5_42	0.00909359	0	Cellulose, hemicellulose & other polymers	Enriched
GH5_5	0.00013329	0	Cellulose, hemicellulose & other polymers	Enriched
GH5_7	0.00113894	0	Cell wall	Enriched
GH88	0.00909359	0	Pectin	Enriched
PL24	0.00909359	0	Sugars	Enriched

Supplementary Table 5, Genes that were selected for validation testing of targeted metabolic pathways. This includes primer sequence, amplicon size, targeted KO and identified in KEGG module with references to its location in *Candida* species.

*Table **Error! No text of specified style in document.**.1*

**Name**	**Gene**	**Accession numbers**	**Primer sequence**	**Amplication size**	**BR KEGG ID**	**KO**	**KEGG Enzyme**	**Gene ID**	**KEGG module**	**Reference**
Onithine decarboxylase, ODC	SPE1	NC_032089.1	CTGATGATTCAACGGCTCAA/TCTTTAGCACCCGATCCAAC	145	cal00330	K01581	EC:4.1.1.17	CAALFM_C100740CA	M00134	PMID: 9392083
Spermidine Synthase	SPE3	NC_032090.1	CTCACCCAAACCCAAAGAAA/TCAATGTCACAAAGCCAAGC	109	cal00330, cal00270	K00797	EC:2.5.1.16	CAALFM_C206960WA	M00133	PMID: 21874756
bifunctional choline kinase/ethanolamine kinase [*Candida albicans SC5314*	CKI1	NC_032091.1	GGTGGAGGAAGAAGGGGTTA/ATGAATTGGGGAGGGCTAGT	147	cal00564	K00866	EC:2.7.1.32	CAALFM_C305300CA	M00090	PMID:13061469
Lysophosphatidylcholine acyltransferase [*Candida albicans SC5314*]	TAZ1	NC_032090.1	AAAAGTGGCTCCCGAAGATT/GTGATCTCCATTGCTCACGA	140	sce01004	K13510	EC:2.3.1.23, 2.3.1.67	YPR140W	-	PMID: 17182612
tetrafunctional fatty acid synthase [Candida albicans SC5314]	FAS1	NC_032093.1	ACCAATTGCACCCATCATTT/CAGGAGTGGTTTCTGGGTGT	84	cal00061 , cal01100 ,cal01212	K00668	EC:2.3.1.86	CAALFM_C500190CA	M00082	PMID:3528750
trifunctional fatty acid synthase subunit [Candida albicans SC5314]	FAS2	NC_032091.1	GTTTGTGGTGCTGTCATTGG/TGAAAGCCATTTCCTTTTGG	121	cal00061 , cal01100 ,cal01212	K00667	EC:2.3.1.86	CAALFM_C304830CA	M00082 ,M00083	PMID:2900835
