# Supplementary material for: Integrative functional analysis uncovers metabolic differences between Candida species
Source: Commun Biol. 2022 Sep 26;5:1013. doi: 10.1038/s42003-022-03955-z (PMC9512779; doi:10.1038/s42003-022-03955-z)
Supplement: Supplementary file 3 — Description of Additional Supplementary Files [file 42003_2022_3955_MOESM3_ESM.pdf]

Description of Additional Supplementary Files

File name: Supplementary Data 1

Description: Fungal species in KEGG database. List of 128 fungal genes in KEGG database, species abbreviation, T number, protein-encoding genes and genes converted to KO.

File name: Supplementary Data 2

Description: The building of BioFung. List of fungal-specific KO available in KEGG database, genes available per KO, missing genes from KO, missing sequences of genes unable to include and failure of multi-sequence alignment in creating BioFung.

File name: Supplementary Data 3

Description: Missing information from the KEGG database. List of genes in fungal species had missing sequences and was unable to collate in profile.

File name: Supplementary Data 4

Description: The categorisation of Pfam clans. Categories were based on clan function to the associated processes; note clans can fit into many functional categories.

File name: Supplementary Data 5

Description: References for Pectin CAZyme assay confirmation. List of publications confirming the function of pectin. Table 9, References for RT-qPCR validation. List of gene selection, primer sequence and accession numbers for gene expression analysis.

File name: Supplementary Data 6

Description: Metabolic pathways enriched in all Candida species from functional annotation of KO and input data to graph from figure 3a.

File name: Supplementary Data 7

Description: Metabolite concentration of AGAu and non-AGAu strains from 40 metabolomics and input data for figure 3c.
