# Supplementary material for: Integrative functional analysis uncovers metabolic differences between Candida species
Source: Commun Biol. 2022 Sep 26;5:1013. doi: 10.1038/s42003-022-03955-z (PMC9512779; doi:10.1038/s42003-022-03955-z)
Supplement: Supplementary file 11 — Reporting Summary [file 42003_2022_3955_MOESM11_ESM.pdf]

nature research

Corresponding author(s): Saeed Shoaie and David Moyes

Last updated by author(s): June 15, 2022

Reporting Summary

Nature Research wishes to improve the reproducibility of the work that we publish. This form provides structure for consistency and transparency in reporting. For further information on Nature Research policies, see our Editorial Policies and the Editorial Policy Checklist.

Statistics

For all statistical analyses, confirm that the following items are present in the figure legend, table legend, main text, or Methods section.

n/a	Confirmed	
☐	☒	The exact sample size (*n*) for each experimental group/condition, given as a discrete number and unit of measurement
☐	☒	A statement on whether measurements were taken from distinct samples or whether the same sample was measured repeatedly
☐	☒	The statistical test(s) used AND whether they are one- or two-sided<br>*Only common tests should be described solely by name; describe more complex techniques in the Methods section.*
☒	☐	A description of all covariates tested
☒	☐	A description of any assumptions or corrections, such as tests of normality and adjustment for multiple comparisons
☐	☒	A full description of the statistical parameters including central tendency (e.g. means) or other basic estimates (e.g. regression coefficient) AND variation (e.g. standard deviation) or associated estimates of uncertainty (e.g. confidence intervals)
☐	☒	For null hypothesis testing, the test statistic (e.g. *F*, *t*, *r*) with confidence intervals, effect sizes, degrees of freedom and *P* value noted<br>*Give P values as exact values whenever suitable.*
☒	☐	For Bayesian analysis, information on the choice of priors and Markov chain Monte Carlo settings
☒	☐	For hierarchical and complex designs, identification of the appropriate level for tests and full reporting of outcomes
☒	☐	Estimates of effect sizes (e.g. Cohen's *d*, Pearson's *r*), indicating how they were calculated

Our web collection on statistics for biologists contains articles on many of the points above.

Software and code

Policy information about availability of computer code

Data collection	Database, code and instructions for use included at: https://github.com/sysbiomelab/BioFung<br>Strains were used from public data available. (included in methods, supplementary table).
Data analysis	Bash was used on High Performance Computing to create BioFung which is publically available and usable with Nextflow (installation instructions included). Data analysis was performed on R version 3.5.0

For manuscripts utilizing custom algorithms or software that are central to the research but not yet described in published literature, software must be made available to editors and reviewers. We strongly encourage code deposition in a community repository (e.g. GitHub). See the Nature Research guidelines for submitting code & software for further information.

Data

Policy information about availability of data

All manuscripts must include a data availability statement. This statement should provide the following information, where applicable:

- Accession codes, unique identifiers, or web links for publicly available datasets
- A list of figures that have associated raw data
- A description of any restrictions on data availability

BioFung is public open access database that can be downloaded at: https://www.microbiomeatlas.org/downloads.php. The instruction and the pipeline scripts for BioFung can be found at our GitHub repository https://github.com/sysbiomelab/BioFung.

Field-specific reporting

Please select the one below that is the best fit for your research. If you are not sure, read the appropriate sections before making your selection.

☒ Life sciences ☐ Behavioural & social sciences ☐ Ecological, evolutionary & environmental sciences

For a reference copy of the document with all sections, see nature.com/documents/nr-reporting-summary-flat.pdf

Life sciences study design

All studies must disclose on these points even when the disclosure is negative.

Sample size	49 strains of Candida species used from previously published data (Pubmed ID enclosed in supplementary tables). 7 clinical derived strains for metabolomics was stated in methods (clinically available for testing representing AGAu and non-AGAu groups.
Data exclusions	No data was excluded. In both genomics and metabolomics all samples were included.
Replication	Experimentally metabolomics samples were taken and qPCR performed in triplicates.
Randomization	There is no randomisation in this paper as clinical derived strains samples were grouped
Blinding	Blinding is not relevant in this study

Reporting for specific materials, systems and methods

We require information from authors about some types of materials, experimental systems and methods used in many studies. Here, indicate whether each material, system or method listed is relevant to your study. If you are not sure if a list item applies to your research, read the appropriate section before selecting a response.

Materials & experimental systems

n/a	Involved in the study
☒	☐ Antibodies
☒	☐ Eukaryotic cell lines
☒	☐ Palaeontology and archaeology
☒	☐ Animals and other organisms
☒	☐ Human research participants
☒	☐ Clinical data
☒	☐ Dual use research of concern

Methods

n/a	Involved in the study
☒	☐ ChIP-seq
☒	☐ Flow cytometry
☒	☐ MRI-based neuroimaging
